# Detrimental Immediate- and Medium-Term Clinical Effects of Right Ventricular Pacing in Patients With Myocardial Fibrosis

**DOI:** 10.1161/CIRCIMAGING.120.012256

**Published:** 2021-05-18

**Authors:** Christopher E.D. Saunderson, Maria F. Paton, Louise A.E. Brown, John Gierula, Pei G. Chew, Arka Das, Anshuman Sengupta, Thomas P. Craven, Amrit Chowdhary, Aaron Koshy, Hazel White, Eylem Levelt, Erica Dall’Armellina, Pankaj Garg, Klaus K. Witte, John P. Greenwood, Sven Plein, Peter P. Swoboda

**Affiliations:** 1 Department of Biomedical Imaging Science, Leeds Institute of Cardiovascular and Metabolic Medicine, University of Leeds, United Kingdom (C.E.D.S., M.F.P., L.A.E.B., J.G., P.G.C., A.D., T.P.C., A.C., A.K., E.L., E.D., K.K.W., J.P.G., S.P., P.P.S.).; 2 Department of Cardiology, Leeds Teaching Hospitals NHS Trust, United Kingdom (A.S.).; 3 Department of Cardiology, Mid Yorkshire Hospitals NHS Trust, Wakefield, West Yorkshire, United Kingdom (H.W.).; 4 Department of Infection, Immunity & Cardiovascular Disease, University of Sheffield, United Kingdom (P.G.).

**Keywords:** atrioventricular block, biomarkers, fibrosis, heart failure, heart ventricles

## Abstract

**Background:**

Long-term right ventricular (RV) pacing leads to heart failure or a decline in left ventricular (LV) function in up to a fifth of patients. We aimed to establish whether patients with focal fibrosis detected on late gadolinium enhancement cardiovascular magnetic resonance (CMR) have deterioration in LV function after RV pacing.

**Methods:**

We recruited 84 patients with LV ejection fraction ≥40% into 2 observational CMR studies. Patients (n=34) with a dual-chamber device and preserved atrioventricular conduction underwent CMR in 2 asynchronous pacing modes (atrial asynchronous and dual-chamber asynchronous) to compare intrinsic atrioventricular conduction with forced RV pacing. Patients (n=50) with high-grade atrioventricular block underwent CMR before and 6 months after pacemaker implantation to investigate the medium-term effects of RV pacing.

**Results:**

The key findings were (1) initiation of RV pacing in patients with fibrosis, compared with those without, was associated with greater immediate changes in both LV end-systolic volume index (5.3±3.5 versus 2.1±2.4 mL/m^2^; *P*<0.01) and LV ejection fraction (−5.7±3.4% versus −3.2±2.6%; *P*=0.02); (2) medium-term RV pacing in patients with fibrosis, compared with those without, was associated with greater changes in LV end-systolic volume index (8.0±10.4 versus −0.6±7.3 mL/m^2^; *P*=0.008) and LV ejection fraction (−12.3±7.9% versus −6.7±6.2%; *P*=0.012); (3) patients with fibrosis did not experience an improvement in quality of life, biomarkers, or functional class after pacemaker implantation; (4) after 6 months of RV pacing, 10 of 50 (20%) patients developed LV ejection fraction <35% and were eligible for upgrade to cardiac resynchronization according to current guidelines. All 10 patients had fibrosis on their preimplant baseline scan and were identified by >1.1 g of fibrosis with 90% sensitivity and 70% specificity.

**Conclusions:**

Fibrosis detected on CMR is associated with immediate- and medium-term deterioration in LV function following RV pacing and could be used to identify those at risk of heart failure before pacemaker implantation.

## Abstract 2

Supplemental Digital Content is available in the text.

**Clinical Perspective**

Long-term right ventricular pacing leads to heart failure or a decline in left ventricular function in up to a fifth of patients. Upfront identification of patients at risk of decline in left ventricular ejection fraction and consequent heart failure after long-term right ventricular pacing remains a clinical challenge. This study showed that the short- and medium-term detrimental effects on left ventricular function during right ventricular pacing are most pronounced in patients with myocardial fibrosis identified by late gadolinium enhancement on cardiovascular magnetic resonance. Myocardial fibrosis may represent a risk factor that could be used prospectively to identify patients susceptible to the detrimental effects of right ventricular pacing. Further work is needed to identify whether patients with fibrosis benefit from alternative pacing strategies or medical therapies applied at the time of the initial implant.

Right ventricular (RV) pacing is an established treatment for symptomatic bradycardia and has been shown to normalize life expectancy and improve quality of life.^1,2^ RV apical pacing has the disadvantage of inducing electrical and mechanical dyssynchrony, which may lead to progressive left ventricular (LV) dysfunction and heart failure.^3–5^ Up to a fifth of patients with a pacemaker for bradycardia will develop heart failure or have a decline in LV ejection fraction (LVEF) with long-term RV pacing.^6,7^ Alternate strategies such as RV septal pacing and pacing avoidance algorithms have failed to improve patient-orientated clinical end points.^8^ The BLOCK-HF trial (Biventricular Pacing for Atrioventricular Block and Systolic Dysfunction) demonstrated improvements in a composite end point of death, heart failure hospitalization, and reduction in LV dimensions with cardiac resynchronization therapy (CRT) over RV pacing in patients with atrioventricular block and an LVEF <50%.^9^ However CRT is associated with a greater upfront economic cost, a higher rate of complications, and shorter battery longevity than dual-chamber pacing, and its utility in patients with preserved or mildly impaired LV function is less established.^10–12^ There is, therefore, a need to improve identification of those at risk of decline in LVEF and consequent heart failure after long-term RV pacing who could benefit from an alternative, personalized upfront pacemaker prescription.

Cardiovascular magnetic resonance (CMR) provides an accurate serial assessment of cardiac volumes, evaluation of LV synchronicity, and detection of myocardial fibrosis.^13^ Late gadolinium enhancement (LGE) imaging is established for the assessment of myocardial fibrosis in a range of cardiovascular conditions. Conventional segmented LGE imaging can be a challenge in bradycardic patients due to the long duration of breath hold required and also in patients with implanted cardiac devices due to off-resonance artifacts.^14^ Recent advances in CMR, and the advent of magnetic resonance imaging (MRI) conditional pacing systems, mean these issues can be overcome using free breathing and wideband LGE pulse sequences.^14^

We aimed to establish whether fibrosis detected on LGE CMR is associated with greater deterioration in LV function following acute and medium-term RV pacing by studying 2 separate patient cohorts.

## Methods

The data that support the findings of this study are available from the corresponding author upon reasonable request.

### Study 1: Immediate Effects of RV Pacing

**Figure 1.**
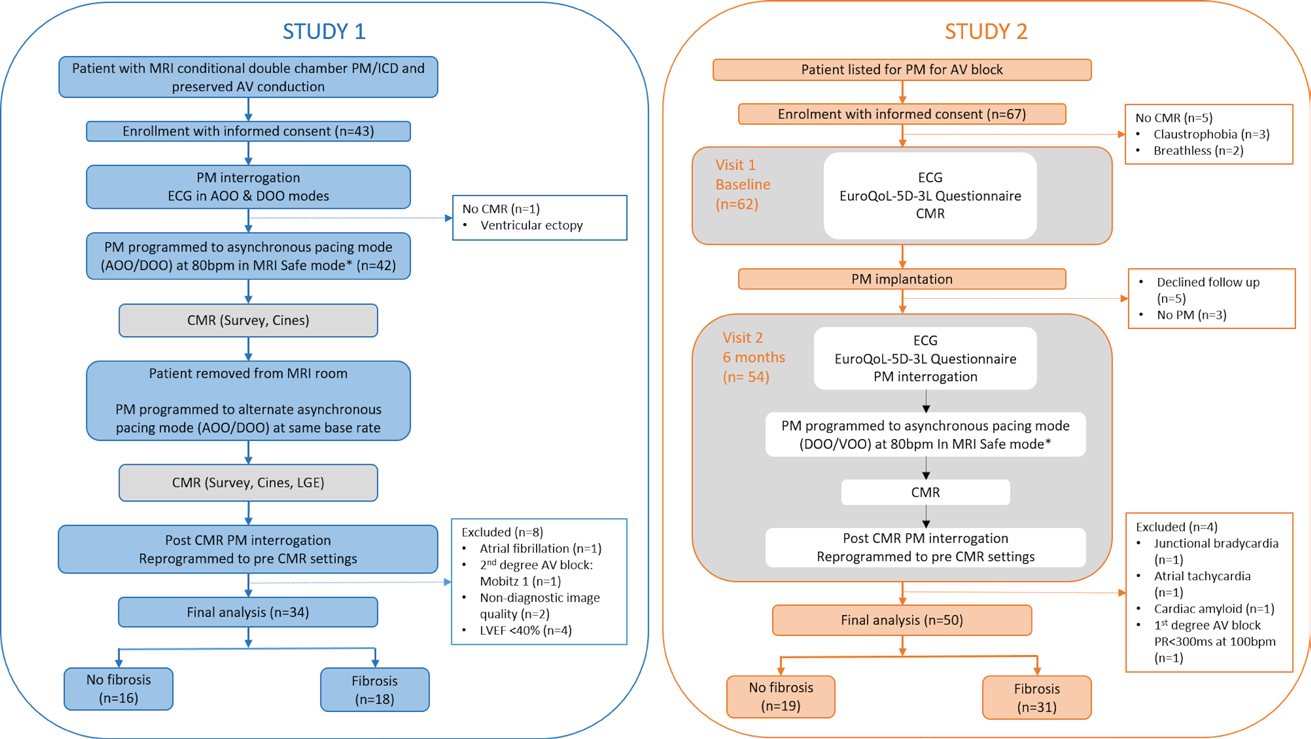
**Study design and protocol.** AOO indicates atrial asynchronous pacing; AV, atrioventricular; CMR, cardiovascular magnetic resonance; DOO, dual chamber asynchronous pacing; ICD, implantable cardioverter defibrillator; LGE, late gadolinium enhancement; LVEF, left ventricular ejection fraction; MRI, magnetic resonance imaging; PM, pacemaker; and VOO, ventricular asynchronous pacing. *Or 10bpm above the intrinsic heart rate when the intrinsic rate was greater than 80bpm.

Forty-three adult patients (>18 years) with dual-chamber MRI conditional pacemakers or secondary prevention implantable cardioverter-defibrillators implanted >6 weeks previously were recruited from the Leeds General Infirmary Pacemaker Service between November 2017 and April 2019. Inclusion in the study required a ventricular pacing burden of <5% and the presence of sinus rhythm at the most recent device interrogation. Exclusion criteria included any contraindication to CMR, pregnancy or breastfeeding, estimated glomerular filtration rate <30 mL/min, and severe valvular heart disease. Patients underwent ECG and a single multiparametric CMR scan, including cine imaging and LGE, in 2 asynchronous pacing modes (atrial asynchronous [AOO] and dual-chamber asynchronous [DOO]) at the same base rate to compare intrinsic atrioventricular conduction with forced dual-chamber pacing. In DOO mode, RV pacing was forced by shortening the paced atrioventricular delay and confirmed with a characteristic QRS pattern on a surface ECG. Patients with reduced LV function (LVEF, <40%) during intrinsic atrioventricular conduction were excluded from the final analysis (Figure 1). Ethical approval was granted by the Health Research Authority (National Research Ethics Service Centre: Yorkshire and Humber REC: 12/YH/0551 and 18/YH/0168). Written informed consent was obtained from all participants.

### Study 2: Medium-Term Effects of RV Pacing

Sixty-seven unselected consecutive adult patients with acquired atrioventricular block scheduled to undergo single- or dual-chamber pacemaker implantation were prospectively recruited from the Leeds General Infirmary between February 2018 and January 2019. Inclusion criteria included class I or IIa indication for a pacemaker due to atrioventricular block and an expectation of a high requirement for RV pacing.^1^ Exclusion criteria included a prior diagnosis of heart failure or LVEF <40%, recent use of temporary pacing system, acute coronary syndrome within the last 30 days, severe valvular heart disease, class 1 CRT indication, and contraindication to CMR. Since all standard pacemaker systems implanted in the Leeds Service are MRI conditional, decisions regarding the indication, type of device, and device programming were taken by the patient’s clinical team. Patients underwent assessment including multiparametric CMR with LGE imaging, before and 6 months after pacemaker implantation (Figure 1). The New York Heart Association functional class and health status, using the EuroQoL-5D-3L questionnaire, were assessed at baseline and follow-up. ECGs and venous blood sampling were performed before CMR from each patient at both visits (Data Supplement). Ethical approval was granted by the Health Research Authority (National Research Ethics Service Centre: East Midlands REC: 17/EM/0475). Written informed consent was obtained from all participants.

#### Power Calculation

In study 2, an a priori power calculation was performed, to detect a clinically meaningful (10 mL) change in LV end-systolic volume between those with and without LV scar based on interstudy SD of 5.4%; a minimum of 7 patients with LV fibrosis determined by LGE were required (α, 5%; β, 10%).^15^ A sample size of 50 was chosen to allow for a minimum prevalence of 14%, which is similar to the prevalence of unrecognized scar in similarly aged populations.^16^

### Device Programming

Before entering the MRI room, patients underwent full device interrogation and were programmed into manufacturer-specific MRI safe mode with tachyarrhythmia therapies disabled in patients with implantable cardioverter-defibrillators.

Patients enrolled into study 1 were programmed to both AOO and DOO pacing, in a random order, at least 10 bpm above their intrinsic heart rate to avoid competition (Figure 1).

In study 2, for the follow-up scan at 6 months, the device was programmed to asynchronous pacing at a base rate of 80 bpm in either ventricular, in patients with a single-chamber pacemaker or in atrial fibrillation, or DOO asynchronous pacing modes. In those with a sinus rate above 80 bpm, the base rate was programmed to 10 bpm above the intrinsic heart rate to avoid competition and ensure sequential atrioventricular pacing.

During the CMR examination, patients were monitored using noninvasive blood pressure and vectorcardiogram signal. A device check was performed immediately after the CMR. Device and lead models are available in Table I in the Data Supplement.

### Cardiovascular Magnetic Resonance

All participants underwent CMR imaging at 1.5T (Ingenia; Philips, Best, the Netherlands), which included cine and LGE imaging. LGE imaging was optimized using free breathing or wideband sequences as required (Data Supplement). All scans were performed with a doctor (C.E.D.S.) and a cardiac physiologist (M.F.P./J.G.) with expertise in cardiac devices in attendance.

### Image Analysis

CMR analysis was performed quantitatively offline by a single operator (C.E.D.S.) blinded to clinical data and scanning order. Analysis was performed using commercially available software (Cvi42; Circle Cardiovascular Imaging, Calgary, Canada). LV volumes and LVEF were calculated by the summation-of-discs method.^15^

**Figure 2.**
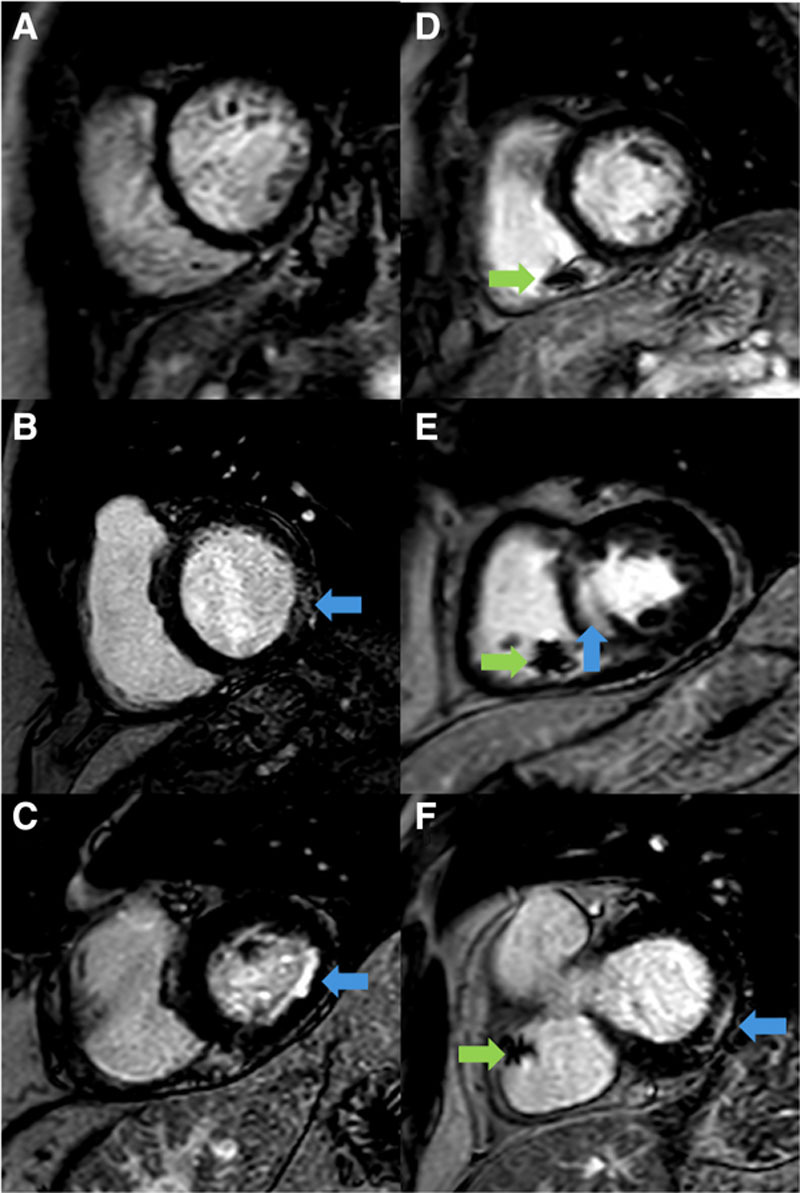
**Late gadolinium imaging in study participants.** Representative short-axis late gadolinium enhancement (LGE) images in bradycardic patients before pacemaker implantation (**A–C**) and in patients with implanted pacemakers (**D–F**). Examples demonstrate the presence of LGE (blue arrows) and artifact from the right ventricular pacing lead (green arrows).

LGE images were visually reviewed for the presence or absence of LGE by at least 2 observers blinded to clinical data (C.E.D.S./L.A.E.B./S.P./P.P.S.; Figure 2). The presence of ischemic and nonischemic patterns of myocardial fibrosis was assessed according to previously published work.^17^ Semiautomated quantification of LGE was then performed using a threshold of 5 SDs of signal intensity of the remote myocardium.^18^ Strain parameters were calculated using feature tracking software (Cvi42) and used to determine a mechanical dyssynchrony index (Data Supplement).

### Biochemical Analysis

NT-proBNP (N-terminal pro-B-type natriuretic peptide) was measured with the Advia Centaur system (Siemens Healthcare Diagnostics, Camberley, United Kingdom), which quantifies NT-proBNP with a range of 35 to 35 000 pg/mL. The intra-assay coefficient of variation was 5% at a concentration of 100 to 500 pg/mL.

### Statistical Analysis

Statistical analysis was performed using SPSS 25 (International Business Machines, Armonk, NY). Continuous variables are expressed as mean±SD or as median with interquartile range and categorical variables, as counts and percentages. Normality for continuous variables was tested using the Shapiro-Wilk test. For normally distributed variables, independent samples *t* testing was used for comparisons between groups and paired samples *t* testing was applied for comparisons within groups. For non-normally distributed variables, independent samples Mann-Whitney *U* test and the related samples Wilcoxon signed-rank test were used as appropriate while comparison of categorical variables used the χ^2^ test. *P*<0.05 was considered significant. Cutoff values of LGE mass at baseline to predict LVEF <35% at 6 months were derived from receiver operator characteristic curve analysis.

## Results

### Study 1: Immediate Effects of RV Pacing

During the prospective data collection period, of the 43 patients recruited, 42 patients underwent assessment, and 8 were excluded leaving 34 patients in the final analysis (Figure 1).

Fibrosis detected by LGE was present in 18 (53%) patients, and differences between those with and without LGE are shown in Table 1. The location and distribution of myocardial fibrosis detected by CMR in study participants is available in Table II in the Data Supplement. Patients with fibrosis were older (74.2 versus 64.2; *P*=0.008), more frequently had a history of myocardial infarction (MI; 56% versus 0%; *P*<0.001), and had greater LV end-diastolic volume index (LVEDVi; 77.5±13.8 versus 65.6±12.4 mL/m^2^; *P*=0.01) and LV end-systolic volume index (LVESVi; 37.6±11.4 versus 29.5±9.8 mL/m^2^; *P*=0.03) without a significant difference in baseline LVEF during intrinsic conduction (52.2±8.0% versus 56.0±6.2%; *P*=0.13).

**Table 1.**
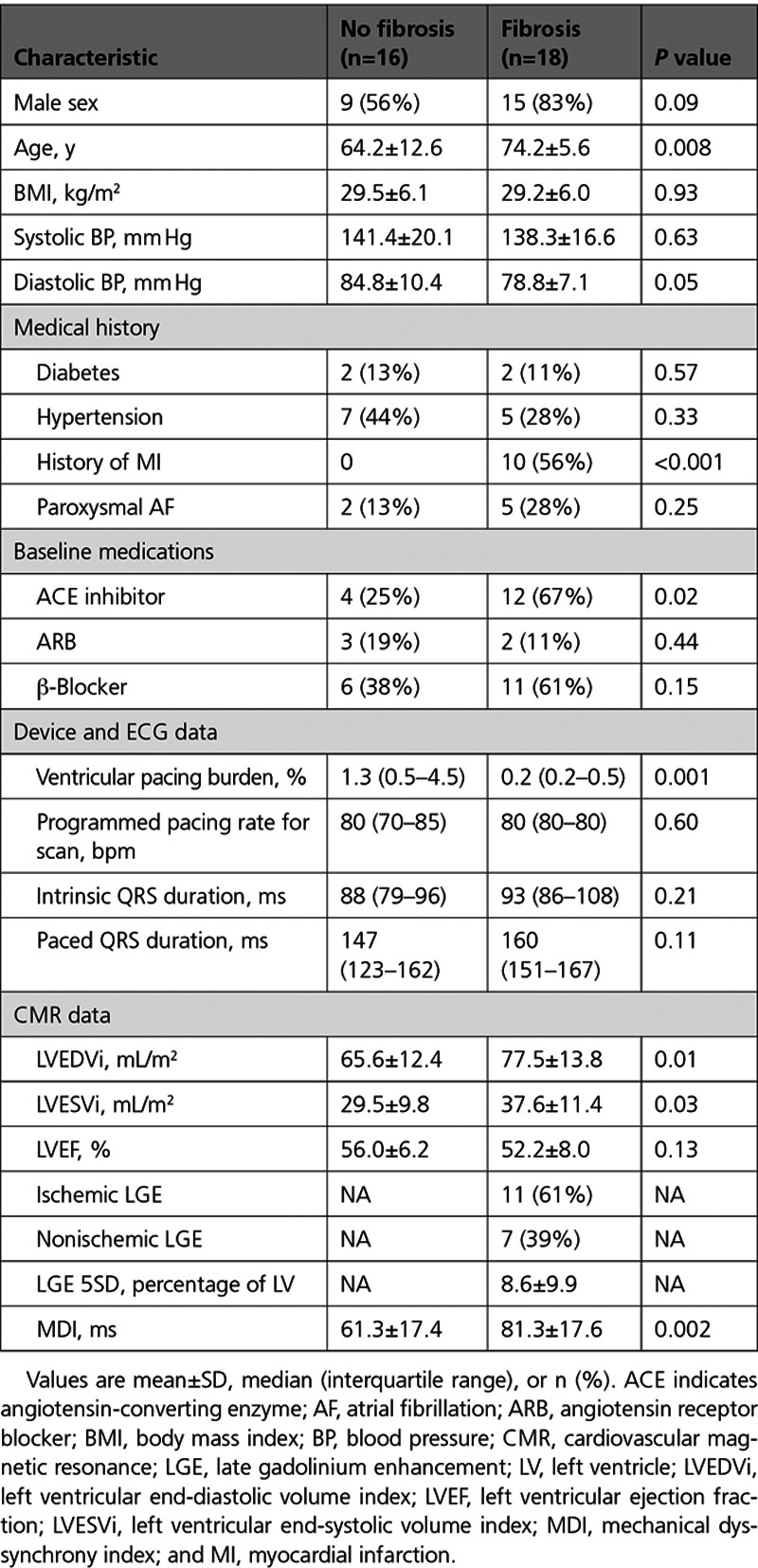
Patient Characteristics and CMR Data in Study 1

**Figure 3.**
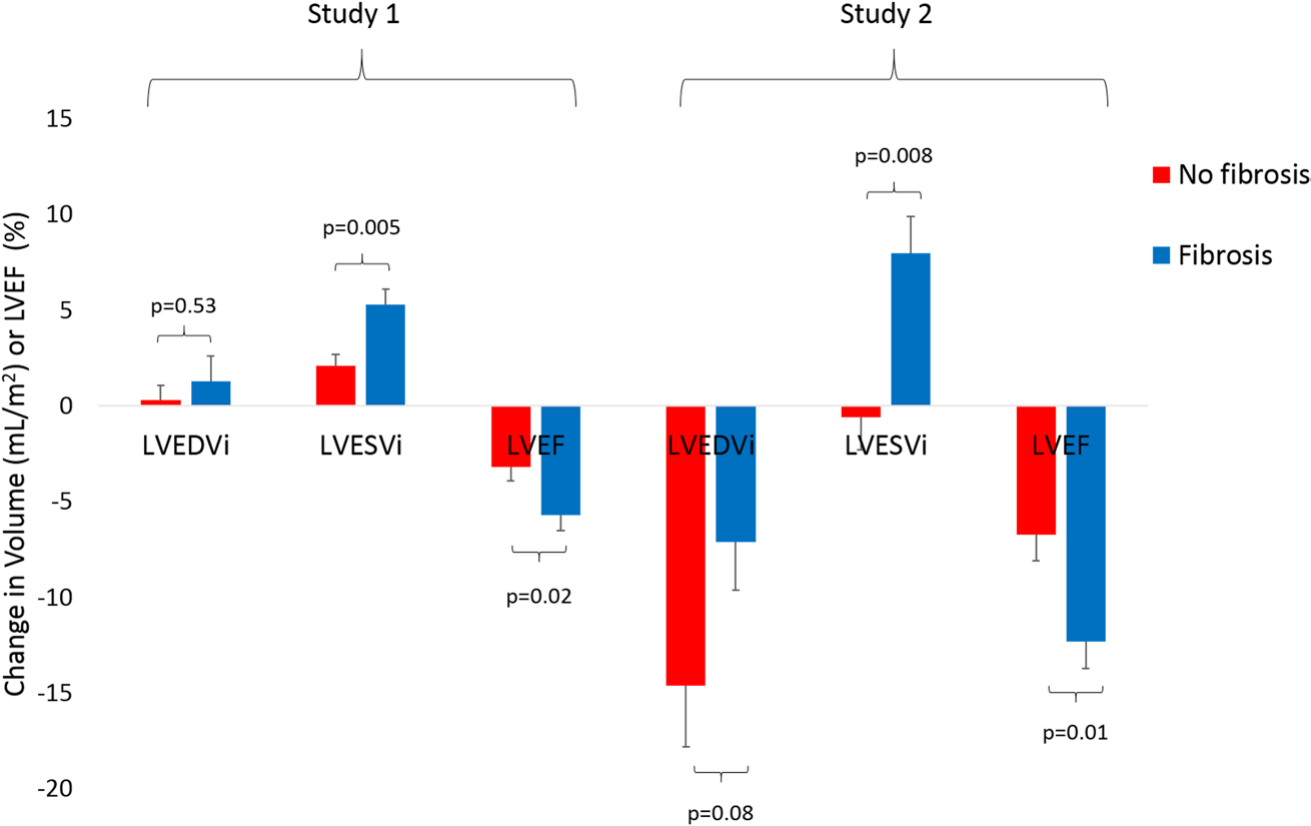
**Absolute change in ventricular volumes and function before and after ventricular pacing.** Values are mean±SE. Absolute changes between those with (blue bars) and without (red bars) myocardial fibrosis. Study 1 (**left**): immediate change between atrial asynchronous and dual chamber asynchronous pacing modes in patients with preserved atrioventricular (AV) conduction. Study 2 (**right**): change from baseline (before pacemaker) to 6 mo follow-up in patients implanted with permanent pacemakers for AV block. LVEDVi indicates left ventricular end-diastolic volume index; LVEF, left ventricular ejection fraction; and LVESVi, left ventricular end-systolic volume index.

DOO pacing was associated with an immediate increase in LVESVi when compared with AOO pacing at the same heart rate, and this change was greater in those with fibrosis than in those without (+5.3±3.5 versus +2.1±2.4 mL/m^2^; *P*=0.005; Table 2; Figure 3). No significant change was observed in LVEDVi between pacing modes in either group. LVEF was lower in both groups during DOO compared with AOO programming but more so in those with fibrosis compared with those without (−5.7±3.4% versus −3.2±2.6%; *P*=0.02). No difference was observed in the intrinsic or paced QRS duration between those with and without fibroses (*P*>0.05). Mechanical dyssynchrony index increased during DOO pacing, compared with AOO pacing, but the change was only statistically significant in patients with fibrosis (88.8±21.2 versus 81.3±17.6 ms; *P*=0.04).

**Table 2.**
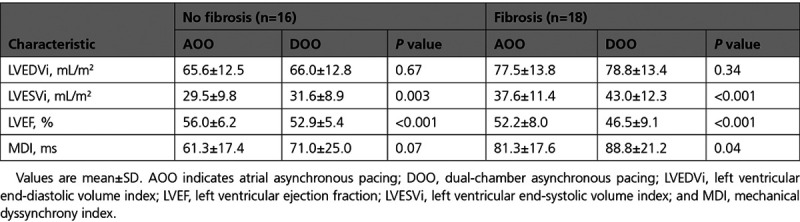
Cardiovascular Magnetic Resonance Data During AOO and DOO Pacing Modes

### Study 2: Medium-Term Effects of RV Pacing

Sixty-seven patients were recruited with 54 completing 6-month follow-up. Four patients were excluded (Figure 1), leaving a total of 50 patients (Table 3). No adverse events secondary to bradycardia were observed during the CMR scan, and imaging was feasible in all patients. Fibrosis by LGE imaging was present in 31 (62%) patients, and the location and distribution of myocardial fibrosis is available in Table II in the Data Supplement.

**Table 3.**
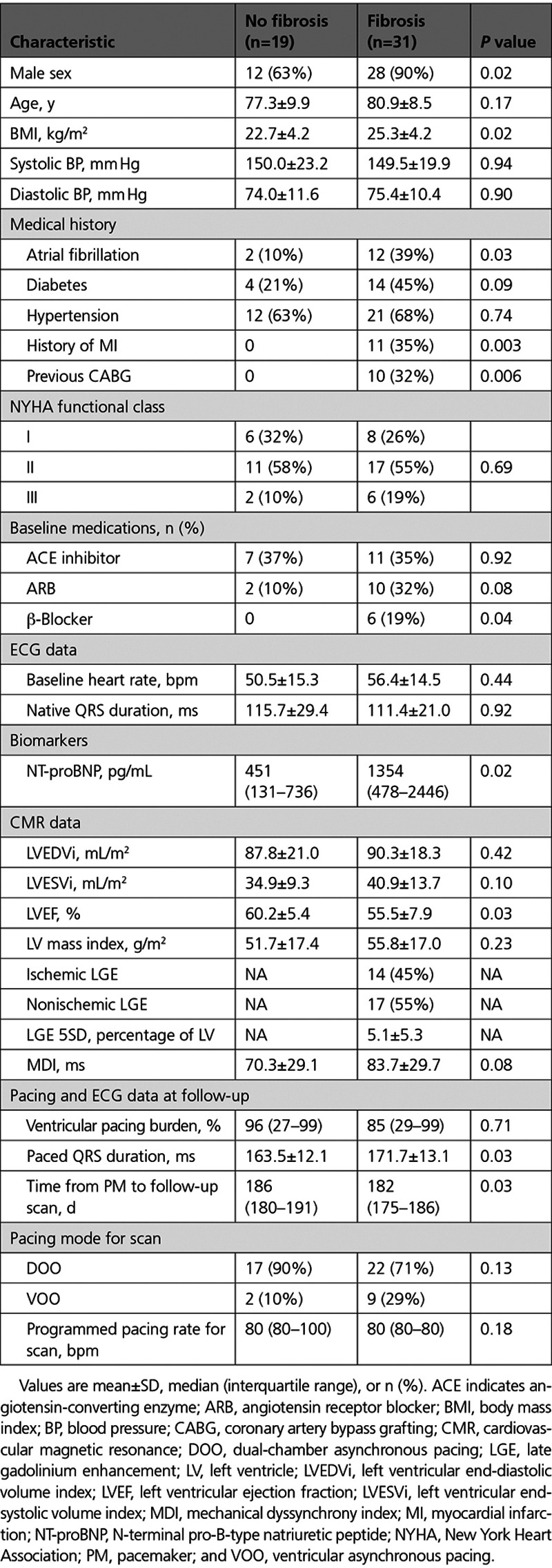
Patient Characteristics and CMR Data in Study 2

Patients with fibrosis were more often male (90% versus 63%; *P*=0.02) and had a higher prevalence of atrial fibrillation (39% versus 10%; *P*=0.03), previous MI (35% versus 0%; *P*=0.003), and previous coronary artery bypass grafting (10% versus 0%; *P*=0.006). No difference was observed in the baseline New York Heart Association class between groups. The distribution of type and severity of conduction tissue disease was not different between those with and without fibrosis (Table III in the Data Supplement). All patients except one had RV leads placed in the RV apex. Patients with fibrosis had higher baseline NT-proBNP (Table 3). Baseline LVEF was lower in those with fibrosis (55.5±7.9% versus 60.2±5.4%; *P*=0.03).

At 6 months, ventricular pacing burdens were the same in patients with and without fibrosis. Patients with fibrosis had a longer paced QRS duration (171.7±13.1 versus 163.5±12.1 ms; *P*=0.03; Table 3). There was a reduction in LVEF in both groups predominantly mediated by a decline in LVEDVi (Table 4). The decline in LVEF was greater in those with fibrosis due to a concomitant increase in LVESVi, which was not seen in those without fibrosis (LVEF: −12.3±7.9% versus −6.7±6.2%, *P*=0.012; LVESVi: 8.0±10.4 versus −0.6±7.3 mL/m^2^, *P*=0.008; Figure 3; Table 4). Patients with fibrosis had a statistically significant increase in mechanical dyssynchrony index from baseline (97.6±31.2 versus 83.7±29.7 ms; *P*=0.03), which was not seen in those without fibrosis. The presence of LGE was an independent predictor of the percentage change in LVESVi after multivariate adjustment (Table IV in the Data Supplement). There was no statistically significant difference in the absolute and percentage of LGE mass (5SD) between baseline and 6-month follow-up (3.7±4.1 versus 3.6±3.6 g, *P*=0.99 and 5.0±5.4% versus 4.6±4.6%, *P*=0.52, respectively).

**Table 4.**
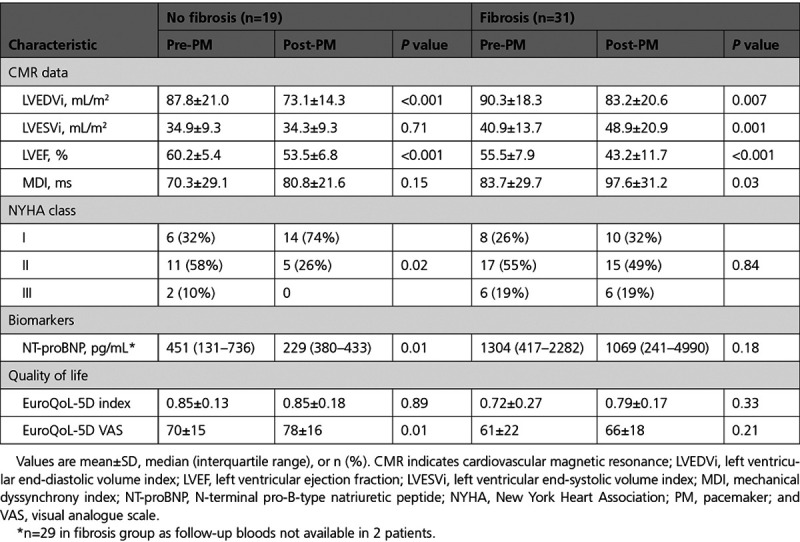
CMR, NYHA Class, and Quality of Life Data Before and After Pacemaker Implantation

Ten patients (20%) had an LVEF of <35% at follow-up and would potentially be eligible for a CRT upgrade according to international guidelines.^1,19^ At baseline, all these patients had an evidence of myocardial fibrosis with mean LVEF of 47.7±5.1%. Six patients had a history of MI, 2 patients had an ischemic pattern of fibrosis with no history of MI, and 2 patients had a nonischemic pattern of fibrosis. On receiver operator characteristic analysis, the area under the curve of fibrosis by 5 SD (g) at baseline to predict LVEF<35% at 6 months was 0.90 (*P*<0.0001 [95% CI, 0.78–0.97]; Figure I in the Data Supplement). By Youden index, the optimal cutoff was >1.1 g fibrosis, which had 90% sensitivity and 70% specificity to predict future eligibility for CRT upgrade.

**Figure 4.**
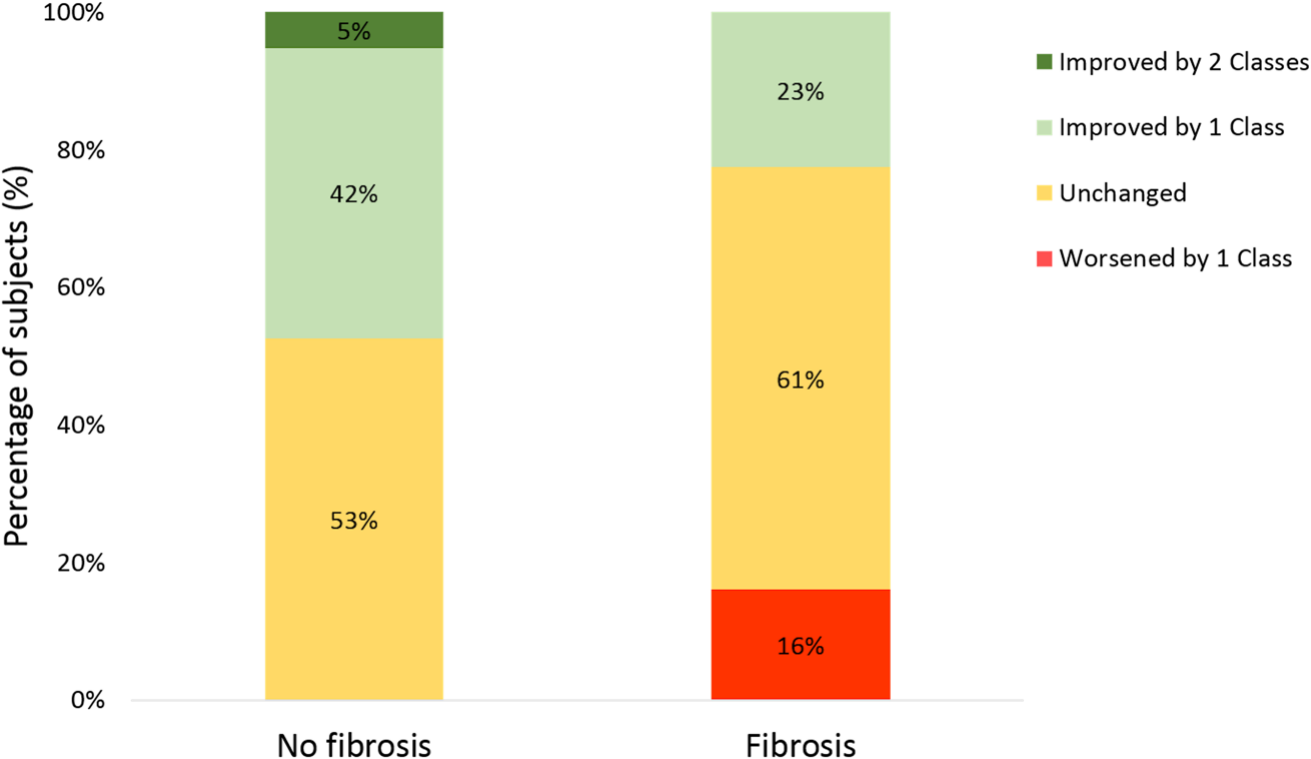
Change in the New York Heart Association functional class before and after pacemaker implantation.

NT-proBNP remained unchanged at follow-up in patients with fibrosis but decreased in those without fibrosis (Table 4). Patients with fibrosis did not experience a statistically significant improvement in the New York Heart Association class or EuroQoL-5D visual analogue scale scores following pacemaker implantation, whereas those without fibrosis had statistically significant improvements in the New York Heart Association class (Figure 4) and EuroQoL-5D visual analogue scale scores.

### Device Parameters

There were no adverse clinical events in either study. In study 1, there were no changes in device variables (Table V in the Data Supplement). In study 2, there were small changes in ventricular lead impedance (722±170 versus 704±150 Ω; *P*=0.006) and ventricular lead pacing capture threshold (0.72±0.26 versus 0.78±0.25 V; *P*=0.005) following the CMR scan.

## Discussion

We have demonstrated that the presence of fibrosis is associated with acute and medium-term reduction in cardiac function following RV pacing with greater electrical and mechanical dyssynchrony and LV remodeling in those with fibrosis. We have also shown that the symptomatic and neurohormonal response, as evaluated by NT-proBNP, to pacemaker implantation in patients with atrioventricular block is reduced in those with fibrosis. Given that fibrosis can be identified before device implantation, it has a potential role in identifying those at risk of LV dysfunction and future heart failure, offering a potential selection criterion for an optimized pacing strategy.

LV dysfunction is common in patients with pacemakers with prevalence as high as 30%.^20^ The mechanisms underlying LV dysfunction in pacemaker recipients are incompletely understood and likely involve complex interactions between factors, such as pacing burden and paced QRS duration, and the underlying myocardial substrate. The fact that LV function can be improved by reducing the burden of RV pacing, through reprogramming or upgrading to CRT in those with unavoidable RV pacing, suggests that pacing-induced dyssynchrony is a key driver of LV remodeling and dysfunction.^21,22^ Furthermore, certain patient populations, such as those with preexisting heart failure or previous MI, may be at greater risk of developing LV dysfunction or heart failure after pacemaker implantation.^7,9,23^ Upfront physiological (CRT or His bundle) pacing may avoid pacing-induced LV dysfunction but comes with higher financial costs and greater risks of complications. Indeed current guidelines do not routinely recommend the use of physiological pacing in those with normal or mildly reduced LVEF.^1,12^ Given that up to a fifth of patients with preserved LVEF develop heart failure or have a decline in LVEF with long-term RV pacing, and the risk may be the greatest within the first 6 months, there is a need for better identification of those at risk before pacemaker implantation.^3,6,7^

### Detrimental Effects of the Combination of Fibrosis and RV Pacing

We have shown that patients with fibrosis had a greater increase in LVESVi and greater decline in LVEF than patients without fibrosis after RV pacing (Figure 3). Compared with intrinsic atrioventricular conduction, the initiation of RV pacing was associated with acute increases in LVESVi and a fall in LVEF. The immediacy of this change likely reflects the acute dyssynchrony induced by RV apical pacing. These changes were more pronounced in patients with underlying myocardial fibrosis. Medium term, those with fibrosis, experienced a greater decline in LVEF through an increase in LVESVi and a reduction in LVEDVi than those without fibrosis. Interestingly those without fibrosis showed no change in LVESVi but also experienced a decline in LVEDVi. We postulate that changes in LVEF due to LVEDVi can be accounted for by differences in heart rate and subsequent LV filling time between baseline and follow-up scans. However medium-term changes in LVESVi only occurred in those with fibrosis and may reflect not only pacing-induced dyssynchrony but also adverse remodeling.

The interaction between differing etiologies of myocardial fibrosis and the susceptibility to pacing-induced LV dysfunction in individuals requires larger scale studies. This may particularly be the case in those with nonischemic pattern of LGE where the mechanisms leading to fibrosis are unclear.

### Clinical Implications

Patients with myocardial fibrosis in this study showed no improvements in either functional class or quality of life after pacemaker implantation in contrast to those without fibrosis. These findings suggest that the observed changes in cardiac function may be clinically significant and may, in part, contribute to the lack of improvement in quality of life and functional capacity. Larger studies with longer follow-up are needed to establish whether RV pacing in patients with myocardial fibrosis is associated with other clinical end points such as heart failure hospitalization and cardiovascular mortality.

Direct comparison of our findings to outcomes of existing clinical trials is challenging due to different study populations, lack of reporting of all echocardiographic findings, and different outcome measures.^8–11^ Furthermore, baseline and follow-up imaging was performed at different time points between studies, and the interaction between heart rate, LVEDV, and LVEF in patients who are profoundly bradycardic potentially makes these volumetric parameters poor for risk stratification preimplantation.

Up to a fifth of patients develop new-onset heart failure or have a decline in LVEF after RV pacing with the highest risk in the first 6 months.^3,6,7^ In our study, 10 patients (20%) had an LVEF of <35% at 6-month follow-up. Despite this, studies of upfront CRT in unselected patients with a high burden of RV pacing have failed to provide clarity. Although in BLOCK-HF, upfront CRT was associated with improved outcomes, the composite end point combined clinical and echocardiographic outcomes in a mixed population, some of whom were indicated for CRT.^9^ In contrast, studies in patients with mostly preserved LV function have shown neutral results.^11^ Therefore, there remains a need for better identification of those at risk of heart failure before pacemaker implantation. The use of CMR upfront enables detection of myocardial fibrosis before pacemaker implantation. In our study, myocardial fibrosis was present in all patients (n=10) who had an LVEF<35% at follow-up who would be eligible for upgrade to CRT. Therefore, the presence of myocardial fibrosis may be an additive risk factor for development of heart failure after RV pacing. Whether long-term clinical outcomes can be improved in those with fibrosis by interventions such as upfront CRT, His bundle pacing, or changes in medical therapy and the cost effectiveness of preimplantation CMR needs to be established.

### Limitations

In both studies, there were statistically significant differences in baseline clinical and imaging characteristics between those with and without fibrosis, which could have influenced intergroup comparisons, and larger studies are warranted to evaluate the impact of these individual parameters on LV function over the longer term.

In study 1, shortening of the atrioventricular delay to encourage RV pacing could have contributed to the observed changes in LVESVi. However, programming of atrioventricular delay was independent of fibrosis status allowing comparison between groups.

In study 2, the type of pacemaker and device programming were managed by the patient’s clinical team. However, clinical teams were blinded to the LGE status minimizing differences in pacing programming between groups. All patients were scanned in an MRI conditional pacing mode mandating ventricular pacing at follow-up to avoid inadvertent inhibition of pacing and to ensure consistency across patients. In patients with a low RV pacing burden, acute dyssynchrony may have been induced and altered LV function. However, the median RV pacing burden was >80% at the prescan check in those with and without fibrosis, making RV pacing in the scanner unlikely to account for the observed differences between the groups.

Neither study was powered to compare differing etiology, degree, or locations of myocardial fibrosis. The influence of quantity and location of fibrosis on RV pacing-induced LV dysfunction needs to be explored in future studies.

### Conclusions

Myocardial fibrosis identified by CMR LGE is associated with immediate- and medium-term impairment in LV function in patients exposed to RV pacing. Myocardial fibrosis may represent a risk factor that could be used prospectively to identify patients susceptible to the detrimental effects of RV pacing. Further work is needed to identify whether patients with fibrosis benefit from alternative pacing strategies or medical therapies applied at the time of the initial implant.

## Acknowledgments

We thank CMR radiographers Gavin Bainbridge, Caroline Richmond, Lisa Lewis, Georgina Casey, Julian Tonge, Faisal Khan, and Margaret Saysell and CMR clinical research nurses Fiona Richards, Hannah Newman, and Petra Bijsterveld for their assistance.

## Sources of Funding

This work was supported by the National Institute for Health Research (NIHR) Leeds Clinical Research Facility. Dr Plein is funded by a grant from the British Heart Foundation, United Kingdom (CH/16/2/32089). The views expressed are those of the authors and not necessarily those of the National Health Service, NIHR, or the Department of Health.

## Disclosures

None.

## Supplementary Material


