# Supplementary material for: Detrimental Immediate- and Medium-Term Clinical Effects of Right Ventricular Pacing in Patients With Myocardial Fibrosis
Source: Circ Cardiovasc Imaging. 2021 May 18;14(5):e012256. doi: 10.1161/CIRCIMAGING.120.012256 (PMC8136461; doi:10.1161/CIRCIMAGING.120.012256)
Supplement: Supplementary file 1 [file hci-14-e012256-s001.pdf]

SUPPLEMENTAL MATERIAL

Supplemental Methods

Storage of blood samples & biochemical analysis

Blood samples were collected in serum separator tubes and centrifuged at 3000 rpm for 10 min within 60 min of collection. Serum was stored at -80 °C prior to analysis.

N-terminal pro-brain natriuretic peptide (NT-proBNP) was measured with the Advia Centaur system (Siemens Healthcare Diagnostics, Camberley, United Kingdom) which quantifies NT-proBNP with a range of 35–35,000 pg/mL. The intra-assay coefficient of variation (CV) was 5% at concentration of 100-500 pg/mL.

Cardiovascular magnetic resonance (CMR) imaging

Scanning took place in normal operating mode (Upper limit of SAR level 2W/kg body weight) with maximised gradient slew rate up to 200T/m/s and according to device manufacturers' instructions. CMR acquisition protocol was as follows

1. Survey images were acquired to identify the cardiac axes.
2. Cine imaging: Acquired using steady state free precession (SSFP) acquisition in a single slice breath-hold sequence. Images obtained included a left ventricular (LV) volume contiguous short axis stack as well as two, three and four chamber views. Typical image parameters were as follows: Slice thickness 10mm, echo time (TE) 1.5 ms, repetition time (TR) 3 ms, flip angle 60°, sensitivity encoding (SENSE) acceleration factor 2 with 30 phases per cardiac cycle.
    a. In Study 1 patients were reprogrammed to the alternate pacing mode as previously described and cine imaging was repeated with identical parameters.

3. If significant susceptibility artefact limiting delineation of endocardial and epicardial borders was present on cine imaging, the patient's ipsilateral arm was positioned above the head where possible to move the susceptibility artefact from the device further from the heart and the scan was repeated.
4. Administration of an intravenous bolus of 0.15mmol/kg gadobutrol (Gadovist®, Bayer, Berlin, Germany) via a cannula followed by a 10ml saline flush.
5. TI scout (Look-locker sequence, single mid-ventricular slice, 10mm thickness, FoV 300x300mm) to determine the optimal TI to null the myocardium
6. Late gadolinium enhanced (LGE) imaging was performed 10-15 minutes after contrast administration:
    a. Study 1
        i. Performed using a T1-weighted phase sensitive inversion recovery (PSIR) gradient echo pulse sequence. Contiguous breath held short axis slices were planned to cover the entire left ventricle (Typically 10-12 slices: same geometry as LV cine imaging). Typical imaging parameters were as follows: 10mm thickness, no interslice gap, matrix 188 x 139, field of view (FoV) 300 × 300 mm, TE 3.0 ms, TR 6.0 ms, flip angle 25°, acquired in-plane resolution 1.60 × 2.15 mm^2reconstructed to 0.89 × 0.89 mm^2, effective SENSE factor 1.8. Two, three and four chamber views were also typically acquired and cross cuts and phase swaps were performed as necessary to confirm the presence or absence of LGE.
        ii. In patients with implantable cardioverter defibrillators (ICDs) a wideband LGE sequence was used to invert off resonant tissue resulting from presence of metal device. A PSIR pulse sequence utilising an increased bandwidth of the inversion pulse (adiabatic pulse of 4 kHz).

No frequency shift was required as no significant artefact remained after utilising the aforementioned parameters.

b. Study 2:

    i. Performed using a T1-weighted PSIR gradient echo pulse sequence. Contiguous breath held short axis slices were planned to cover the entire left ventricle (Typically 10-12 slices: same geometry as LV cine imaging). Typical imaging parameters were as follows: 10mm thickness, no interslice gap, matrix 188 x 139, FoV 300 × 300 mm, TE 3.0 ms, TR 6.0 ms, flip angle 25°, acquired in-plane resolution 1.60 × 2.15 mm^2reconstructed to 0.89 × 0.89 mm^2, effective SENSE factor 1.8. Two, three and four chamber views were also typically acquired and cross cuts and phase swaps were performed as necessary to confirm the presence or absence of LGE.

    ii. If patients were unable to complete breath holds then patients were instructed to free-breathe and a respiratory echo-based navigator was placed on the right hemi diaphragm with a gating window of 6mm with continuous gating level drift activated.

    iii. If the above steps were unsuccessful at obtaining diagnostic images then a single shot inversion recovery SSFP sequence was performed. Typical imaging parameters were as follows: 10mm thickness, no interslice gap, matrix 192 x 144, TE 2.1 ms, TR 4.4 ms, flip angle 20°, acquired in-plane resolution 1.82 × 2.44 mm^2 reconstructed to 1.22 × 1.22 mm^2, effective SENSE factor 2.

Cardiovascular magnetic resonance (CMR) imaging analysis

Strain parameters were calculated using feature tracking software (Cvi42, Circle Cardiovascular Imaging, Calgary, Canada) from the short axis LV and 2-, 3- and 4-chamber cine acquisitions. Epi- and endocardial borders were traced manually at end-diastole and the software then tracked the voxel features of the myocardium to quantify myocardial motion and calculate strain values. Basal slices with through-plane distortion of the LV outflow tract during the cardiac cycle and apical slices with no clear blood pool in systole were not analysed. The time to peak radial strain was derived for all segments of the American Heart Association 16 segment model. Mechanical dyssynchrony index (MDI) was calculated from the standard deviation of the time to maximum radial strain (ms) for the 16 segments.

Supplemental figures and figure legends

Supplemental Figure 1

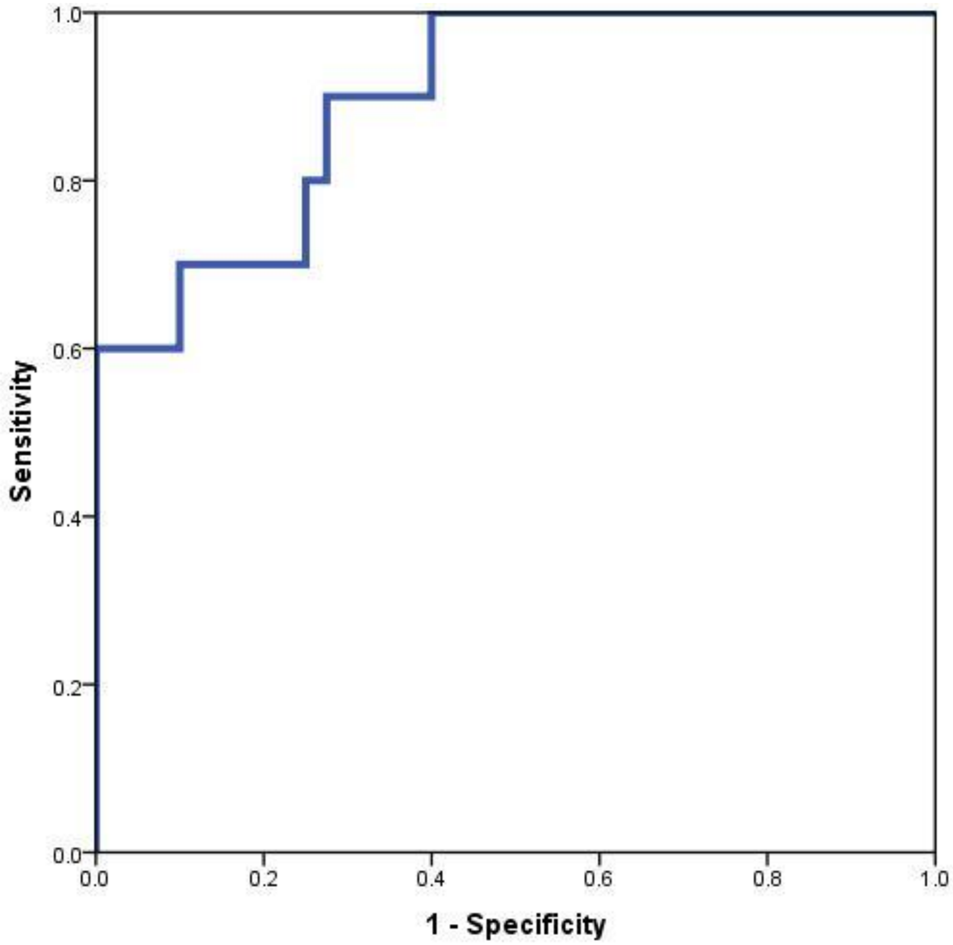

Receiver Operator Characteristic curve for late gadolinium enhanced mass at baseline (5SD [g]) to predict LVEF< 35% at 6 month follow-up.

Supplemental Tables

Supplemental table I. Device and lead manufacturers and models

Manufacturer	Model	Study 1 (n=34)	Study 2 (n=50)
**IPG/ICD**			
**Boston Scientific®**	Essentio SR L110	0	4 (8%)
	Ingenio DR J177	1 (3%)	0
	Proponent DR EL231	9 (26%)	23 (46%)
**Medtronic®**	Ensura SR EN1SR01	0	1 (2%)
	Ensura DR EN1DR01	6 (18%)	0
	Advisa DR A3DR01	5 (15%)	0
	Azure W3DR01	1 (3%)	4 (8%)
	Evera MRI S DR DDMC3D4	4 (12%)	0
**St Jude Medical®**	Assurity MRI PM2272	3 (9%)	1 (2%)
	Endurity MRI PM1172	0	4 (8%)
	Endurity MRI PM2172	5 (15%)	13 (26%)
**Lead**			
**Boston Scientific®**	Ingevity MRI (7731, 7732, 7735, 7736, 7740, 7741, 7742)	20 (29%)	50 (55%)
**Medtronic®**	Capsure Fix (5076)	22 (32%)	6 (7%)
	Capsure Sense (4074, 4574)	6 (9%)	3 (3%)
	Sprint Quattro Secure (6935M, 6947M)	4 (6%)	0
**St Jude Medical®**	Isoflex (1944, 1948)	6 (9%)	9 (10%)
	Tendril MRI (LPA1200M)	2 (3%)	0
	Tendril STS (2088TC)	8 (12%)	23 (25%)

Values are n (%).

ICD models are presented in *italics*

Abbreviations: IPG = Implantable pulse generator; ICD = Implantable cardioverter defibrillator.

Supplemental table II. Location and distribution of myocardial fibrosis detected by CMR in study participants.

	Study One (n=18)	Study Two (n=31)
**Ischaemic pattern**	**n=11**	**n=14**
LAD	7 (64%)	4 (29%)
Cx	2 (18%)	1 (7%)
RCA	2 (18%)	9 (64%)
**Non-ischaemic pattern**	**n=7**	**n=17**
**Midwall**		
Septum	3 (43%)	13 (76%)
Lateral	3 (43%)	3 (18%)
Inferior	0	1 (6%)
**Epicardial**		
Lateral	1 (14%)	0

Values are n (%).

Abbreviations: Cx = circumflex; LAD = left anterior descending; RCA = right coronary artery.

Supplemental table III. Indication for device implantation and right ventricular lead positon.

Characteristic	No fibrosis	Fibrosis	p-value
**Study 1**	**n=16**	**n=18**	
**Indication for device**			
Sinus node disease	12 (75%)	11 (61%)	
AV block	4 (25%)	3 (17%)	0.14
Secondary prevention ICD	0	4 (22%)	
**Ventricular lead position**			
Apex	16 (100%)	18 (100%)	NA
**Study 2**	**n=19**	**n=31**	
**Indication for device**			
Type 1 AV block	2 (10%)	4 (13%)	
Type 2 AV block (Mobitz 1)	2 (10%)	4 (13%)	0.95
Type 2 AV block (Mobitz 2)	7 (37%)	9 (29%)	
Type 3 AV block	8 (43%)	14 (45%)	
**Ventricular lead position**			
Apex	18 (95%)	31 (100%)	
Septum	1 (5%)	0	0.12

Values are n (%).

Abbreviations: AV = atrioventricular; ICD = implantable cardioverter defibrillator

Supplemental table IV. Univariate and multivariate analysis of percentage change in LVESVi.

	Coefficient B	Standard Error	P Value	Coefficient B	Standard Error	P Value
	Univariate analysis -% change in LVESVi			Multivariate analysis -% change in LVESVi		
**LGE**	16.279	5.265	0.003	13.461	5.421	0.017
**LVEF**	-0.577	0.374	0.130			
**Baseline QRS duration**	-0.033	0.116	0.777			
**Paced QRS duration**	0.207	0.211	0.331			
**Age**	0.164	0.309	0.597			
**Gender**	-2.640	6.986	0.707			
**History of AF**	14.434	5.874	0.018	10.001	5.860	0.094
**Ventricular pacing %**	-0.087	0.073	0.243			

Abbreviations: AF = atrial fibrillation; LGE = late gadolinium enhancement; LVEF = left ventricular ejection fraction

Supplemental table V. Device parameters before and after CMR.

Parameter	Study 1			Study 2		
	**Pre CMR value (n=34)**	**Post CMR value (n=34)**	**p-value**	**Pre CMR value (n=50)**	**Post CMR value (n=50)**	**p-value**
**Pacing lead impedance - Ohms**						
Atrial lead	487 (437-555)	489 (439-551)	0.24	513 (430-693)	586 (430-666)	0.96
Ventricular lead	570 (488-749)	588 (460-692)	0.12	695 (589-772)	688 (551-746)	<0.01
**Pacing lead threshold – V@0.4ms**						
Atrial lead	0.5 (0.5-0.8)	0.6 (0.5-0.8)	0.49	0.7 (0.5-0.875)	0.7 (0.6-0.9)	0.57
Ventricular lead	0.8 (0.5-1.0)	0.8 (0.5-1.0)	0.40	0.625 (0.5-0.8)	0.75 (0.58-1.00)	<0.01
**Battery Voltage* - V**	3.02 (3.01-3.02)	3.02 (3.01-3.02)	NA	3.02 (3.01-3.04)	3.02 (3.01-3.04)	NA
**P-wave amplitude - mV**	3.8 (2.5-4.8)	3.8 (2.4-5.0)	0.86	3.9 (3.1-6.6)	4.5 (3.4-5.8)	0.80
**R-wave amplitude - mV**	12 (7.4-15.3)	12 (7.9-16.5)	0.67	14.9 (10.0-21.4)	15.4 (10.9-22.0)	0.55

Values are median (interquartile range). *Boston Scientific® devices were excluded as the programmer does not given a numerical value for battery voltage (n=8 for Study 1 and n=23 for Study 2).

Abbreviations: CMR = Cardiovascular magnetic resonance
